# Supplementary material for: Calpain Regulates Reactive Oxygen Species Production during Capacitation through the Activation of NOX2 and NOX4
Source: Int J Mol Sci. 2023 Feb 16;24(4):3980. doi: 10.3390/ijms24043980 (PMC9967964; doi:10.3390/ijms24043980)
Supplement: Supplementary file 1 [file ijms-24-03980-s001.zip › ijms-2098339-supplementary.pdf]

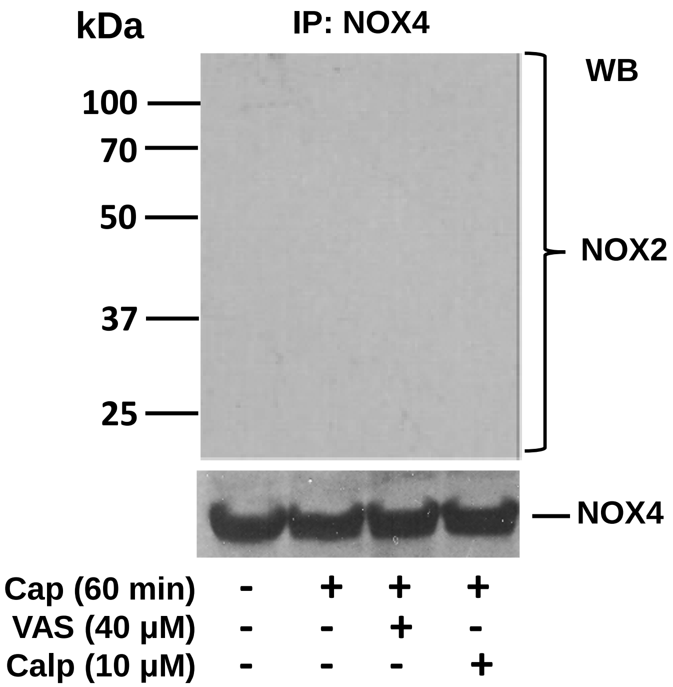

Supplementary Figure S1. Negative control of the coimmunoprecipitation. By Wb was determined that none of the tested proteins was associated non-specifically with the agarose-protein A/G-IgG beads.
